# Increased freshwater discharge shifts the trophic balance in the coastal zone of the northern Baltic Sea

**DOI:** 10.1111/j.1365-2486.2012.02718.x

**Published:** 2012-05-17

**Authors:** Johan Wikner, Agneta Andersson

**Affiliations:** * Department of Ecology and Environmental Science, Umeå UniversitySE-901 87, Umeå, Sweden; † Umeå Marine Sciences Centre, Umeå UniversitySE-910 20, Norrbyn, Hörnefors, Sweden; ‡ The Swedish Institute for the Marine Environment, Unit at Umeå UniversityNorrbyn, SE-910 20, Hörnefors, Sweden

**Keywords:** bacterioplankton, climate, DOC, food web, growth, marine, nutrient, phytoplankton, precipitation

## Abstract

Increased precipitation is one projected outcome of climate change that may enhance the discharge of freshwater to the coastal zone. The resulting lower salinity, and associated discharge of both nutrients and dissolved organic carbon, may influence food web functioning. The scope of this study was to determine the net outcome of increased freshwater discharge on the balance between auto- and heterotrophic processes in the coastal zone. By using long-term ecological time series data covering 13 years, we show that increased river discharge suppresses phytoplankton biomass production and shifts the carbon flow towards microbial heterotrophy. A 76% increase in freshwater discharge resulted in a 2.2 times higher ratio of bacterio- to phytoplankton production (P_b_:P_p_). The level of P_b_:P_p_ is a function of riverine total organic carbon supply to the coastal zone. This is mainly due to the negative effect of freshwater and total organic carbon discharge on phytoplankton growth, despite a concomitant increase in discharge of nitrogen and phosphorus. With a time lag of 2 years the bacterial production recovered after an initial decline, further synergistically elevating the microbial heterotrophy. Current climate change projections suggesting increased precipitation may therefore lead to increased microbial heterotrophy, thereby decreasing the transfer efficiency of biomass to higher trophic levels. This prognosis would suggest reduced fish production and lower sedimentation rates of phytoplankton, a factor of detriment to benthic fauna. Our findings show that discharge of freshwater and total organic carbon significantly contributes to the balance of coastal processes at large spatial and temporal scales, and that model's would be greatly augmented by the inclusion of these environmental drivers as regulators of coastal productivity.

## Introduction

Climate scenarios suggest that the increased CO_2_ levels in the atmosphere and associated warming will increase precipitation in the northern hemisphere (Dore, b^2005^). For northern Scandinavia, precipitation is predicted to increase by a maximum of 21% compared to the current average (Meier, b^2006^). As a result the discharge of freshwater, and thereby nutrient load to the coastal zone, will increase.

The current view is that increased discharge of nitrogen and phosphorus promotes phytoplankton productivity and therefore eutrophication in the coastal zone (Larsson *et al*., b^1985^; Smith, b^2006^; Finkel *et al*., b^2010^). This is based on the fact that most studies implicate phosphorus and nitrogen as the major limiting nutrients of phytoplankton biomass production (Rabalais *et al*., b^2002^). Consequently, increased fresh water discharge should result in elevated primary production and high food web efficiency, and so in greater fish and shellfish production (Nixon, b^1988^).

However, Howarth *et al*. (b^2000^), contrastingly reported higher primary production during dry than wet years in the Hudson estuary, suggesting weaker stratification and light penetration to be the main cause. Also, organic carbon is typically discharged at higher levels simultaneously with nitrogen and phosphorus, potentially exerting several negative effects on phytoplankton productivity (Hessen *et al*., b^2010^). In accordance with this, estuaries worldwide are often reported as net-heterotrophic, suggesting that externally supplied organic carbon is an important driver of coastal metabolism (Kemp *et al*., b^1997^; Sandberg *et al*., b^2004^). Dissolved organic carbon may support bacterioplankton biomass production, resulting in increased competition for mineral nutrients with phytoplankton (Pengerud *et al*., b^1987^; Thingstad *et al*., b^2008^; Barrera-Alba *et al*., b^2009^). Increased freshwater discharge may further influence stratification of the water column, affecting the vertical distribution of phytoplankton and their effective light climate (Cole *et al*., b^1992^; Howarth *et al*., b^2000^; Jager *et al*., b^2008^). Furthermore, an increase in humic and suspended substances may reduce light climate and, in conjunction with reduced salinity, may change the taxonomic composition of resident communities (Gasiunaite *et al*., b^2005^; Hessen *et al*., b^2010^).

Marine productivity and transfer efficiency in the coastal food web are important to understand in view of changing freshwater discharge, as they may influence the functionality of the coastal ecosystem. Whether larger phytoplankton or small bacterioplankton dominate the biomass at the food web base will influence the food web efficiency, defined as fish production per unit of primary production (Nixon, b^1988^; Rand & Stewart, b^1998^; Berglund *et al*., b^2007^). If the net effect of increased freshwater discharge is higher primary production of larger phytoplankton in the coastal zone, this may lead to increased production of fish and shellfish (Finkel *et al*., b^2010^). In contrast, the large influence of riverine dissolved and suspended organic matter in the coastal zone can both reduce primary production and promote a microbial food web structure with poor transfer efficiency of organic carbon to fish and shellfish (Sandberg *et al*., b^2004^; Berglund *et al*., b^2007^). To properly manage coastal resources and mitigate future effects of climate change, a better understanding of the net effect of increased freshwater discharge on an ecosystem scale is therefore required.

Taken together, it is difficult to predict the net outcome of increased river discharge for coastal productivity and the balance between auto- and heterotrophic processes, or to simulate it in controlled laboratory experiments. In this study, we therefore investigated the influence of a relatively moderate, but climatologically relevant, change in freshwater discharge on the ratio of bacterial to phytoplankton biomass production in full-scale coastal ecosystems over several years. This was used as a proxy for the relative importance of microbial production, and thereby losses from fish production and sedimentation.

Three different coastal sea areas were included, providing varying riverine load, morphology, productivity level and hydrography. In addition, long-term effects were examined by using a 13 year data set. The advantage of this strategy was that it addressed many of the composite effects of increased freshwater discharge on hydrography, chemistry, and biology in the coastal zone. Long-term field data sets also encompass effects on larger and more relevant time scales than can be achieved in controlled experimental systems, an important aspect in assessing climate-driven environmental change.

## Materials and methods

### Sampling stations

**Fig. 1:**
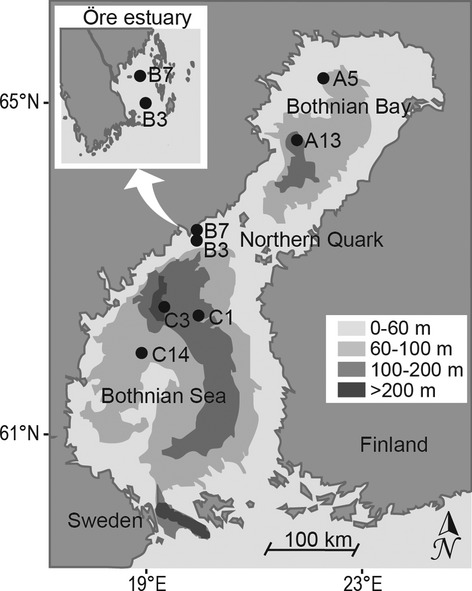
The figure shows the study area of the northern Baltic Sea, with sampling stations, basins, and bottom topography. The Bothnian Bay and Bothnian Sea are divided by the northern Quark. The Öre estuary is shown in the inset map.

The study was performed between 1994 and 2006 in three different basins within the northern Baltic Sea that have varying freshwater load, size, and maximum depth (Table 1; Fig. 1). Bothnian Bay is divided from the Bothnian Sea by the 20 m deep northern Quark (Wulff *et al*., b^1990^). The Öre estuary is relatively open towards the off-shore Bothnian Sea and lacks a marked sill. The catchment area consists mainly of coniferous forests and mires, and a smaller proportion of mountainous areas with tundra vegetation (Sweden) and arable land (Finland).

**Table 1:** Characteristics of sampling stations and basins are shown. Mean load of riverine total organic carbon (TOCr) 1994–2006 is presented

Basin	Area (km^2^)	Volume (km^3^)	Maximum depth (m)	Mean depth (m)	Water exchange (yr)	Load of TOC (10^6^ kg yr^−1^)
Bothnian Bay (Ignatius *et al*., b^1981^)	36, 800	1490	147	43	5	329
Öre estuary (Brydsten, b^1992^)	50	1	–	16	0.03	12
Bothnian Sea (Ignatius *et al*., b^1981^)	66, 000	4340	294	68	3	483

Between 13 and 26 samplings per year and basin were performed from a research vessel, allowing production rate measurements and sample preparation typically to start within 1 h from sampling. From 1994 to 1999, one station per basin was visited, while sampling was extended to include two replicate stations in each basin (about 70 km apart) after the year 2000. Samples were taken with 7 dm^3^ Niskin® bottles (General Oceanics Inc., Miami, FL, USA) mounted on a rosette sampler. Sampling stations in the Bothnian Bay were A5 (65°10.00N; 23°14.00E) and A13 (64°42.50N; 22°4.00E); in the Bothnian Sea, C1 (62°35.20N; 19°58.41E), C3 (62°39.17N; 18°57.14E), and C14 (62°5.99N; 18°32.91E); and in the Öre Estuary station, B3 (63°29.98N; 19°49.14E) and B7 (63°31.50N; 19°48.49E). Station C3 replaced station C1 from the year 2000 an onwards: both represented the north-western Bothnian Sea. Samplings were reduced to 6 yr^−1^ at station C1 1998, due to a temporary reallocation of funding.

### Phytoplankton biomass production (P_*p*_)

Until the year 2000, water samples were taken at distinct depths and incubated at the sampling depth, attached to a drifting buoy (Andersson *et al*., b^1996^). CO_2_ fixation was measured at five depths by uptake of H^14^CO_3_^−^ (2.1 mCi mmol^−1^; Danish Hydrological Institute®) by phytoplankton during 3 h incubation periods, as described in Steeman-Nielsen (b^1952^). Depths used were 1, 2, 5, 10, and 20 m. Daily CO_2_ fixation was calculated by multiplying with the quotient of integrated diel light irradiance and irradiance during the incubation. Light irradiance in water was measured with a Li-Cor LI-193SA spherical sensor (Li-Cor Corporate Offices, Lincoln, NE, USA) and surface irradiance with a Li-Cor LI-190SA planar sensor (400–700 nm). Trapezoidal integration was used to calculate integrated values. From 2001, samples were collected with a 10 m hose and incubation was undertaken onboard the research ship in an ICES incubator with differently shaded incubation bottles (Riegman *et al*., b^1990^). Photosynthetic maximum (P_max_) and maximum light utilization coefficient (*α*_P:I_) was calculated by fitting data to a tangential equation according to Jassby & Platt (b^1976^). Daily CO_2_ fixation was calculated from the parameters P_max_, *α*_P:I_, surface reflection of light (empirically derived 0.82) and hourly mean irradiance at given depths from light attenuation and irradiance below the sea surface. Values down to 20 m were modelled using calculated light irradiance at each depth and assuming estimated coefficients above 10 m to be applicable. Light attenuation was determined by fitting ln transformed data to Beer's law, taking deviating surface values, ship shadowing, and nonlinear data into account. Trapezoidal integration was used to calculate integrated values. Phytoplankton specific growth rate, r_phyto_, was calculated as the ratio of P_p_ and biomass of phytoplankton. This was measured by the Uthermöl technique in Lugol preserved samples, using a sedimentation chamber according to Andersson *et al*. (b^1996^).

A calibration of the different incubation strategies showed no significant differences, according to a Model II regression of ln transformed data (correction factor = 1.08, 95% CI ± 0.27, *n* = 61); neither was a level shift in the time series observed at the time of method shift (Fig. S1).

### Bacterioplankton biomass production (P_b_)

Bacterioplankton biomass production rate was measured by uptake of ^3^H-Thymidine (85 Ci mmol^−1^; Amersham®; GE Healthcare, Buckinghamshire, UK) in 1.5 mL Eppendorf tubes (Smith & Azam, b^1992^). Thymidine uptake in natural samples during short incubations (1 h) in small volumes (1 mL) is specific for active heterotrophic bacteria, as shown by microautoradiography (Fuhrman & Azam, b^1982^). Thymidine uptake was converted to cells produced by an empirically derived factor of 1.4 × 10^18^ cells [mol ^3^H-Thymidine]^−1^ (±SE 0.1 × 10^18^, *n* = 73), calibrating the thymidine uptake to growth of the natural bacterial community (Wikner & Hagström, b^1999^). The carbon density per cell was determined by image analysis of acridine orange-stained cells and the published volume-to-carbon density functions (Norland, b^1993^; Blackburn *et al*., b^1998^). The ratio of bacterio- to phytoplankton biomass production rate was defined as the P_b_:P_p_ ratio.

### Precipitation

Data on precipitation was provided from the Swedish Meteorological and Hydrological Institute's regular monitoring program (SMHI, b^2009^). The station in Haparanda (lat. 65.84, long. 24.11) was representing the Bothnian Bay and Sundsvall airport (lat. 62.52, long. 17.44) represented the Bothnian Sea.

### Riverine flow rate

Data on river flow was provided from the Swedish Meteorological and Hydrological Institute's regular monitoring program (SMHI, b^2009^). The hydrological database is generally based on daily (24 h) data of water flow (m^3^ s^−1^). In the studied sea area 157 stations, covering 30 major rivers in the drainage area, contributed to the drainage basin aggregated flow values. Water flow was measured by water-level gauges calibrated by current measurements in river cross-sections (Anonymous, b^1979^; Bergstrom & Carlsson, b^1994^). For smaller watercourses, artificial measuring weirs were used, and discharge calculated by hydraulic formulas. Monthly means have a reported error of ±5%.

### Discharged total organic carbon, total nitrogen, and total phosphorus

Total organic carbon (TOC_r_), nitrogen (TN_r_), and phosphorus (TP_r_) in river water were measured by the Swedish University of Agricultural Sciences, using standardized protocols within the Swedish monitoring program for river run-off (Demandt, b^2009^). Within this monitoring program, 25 major rivers in the drainage basins were measured at a single sampling station per river, per month. TOC_r_ was analysed in HCL-acidified samples by high-temperature catalytic oxidation with a Shimadzu TOC_r_ 5050® instrument (Peltzer *et al*., b^1996^). Total nitrogen and phosphorus were determined by oxidative digestion with peroxodisulfate and spectrophotometric analysis in a Technicon Autoanalyzer (Bran & Luebbe, Solna, Sweden), according to the International Organization for Standardization (ISO) 11905-1 and ISO 6878, respectively.

### Load of riverine organic carbon to the sea

The discharge of total organic carbon (TOC_r_) was normalized to the sea basin area to harmonize with biomass production variables in units per area (Table 1). The TOC_r_ was assumed to be uniformly distributed in each basin over the course of a year. For the Öre estuary, 10% of the TOC_r_ was assumed to be metabolized in the estuary, as 90% has been shown to be exported to the off-shore areas of the Bothnian Sea (Forsgren & Jansson, b^1992^). Other sources of TOC_r_ to the marine environment were assumed to be negligible (Sandberg *et al*., b^2004^).

### Data aggregation

Values from distinct depths were integrated by the trapezoidal method over the average water column in each basin (Table 1). Carbon dioxide fixation values were integrated to 20 m. Where measurements were not available to maximum depths, this value was extrapolated from closest relevant depths and stations to minimize integration differences. Yearly productivity values were calculated from daily values by using trapezoidal integrations.

### Statistics

Data was tested for normal distribution by the Shapiro–Wilks test, and for homogeneity of variances according to the Levene statistic, using SPSS® statistical software (v. 18; IBM Corporation, Armonk, NY, USA). Values were transformed when data differed from normal distribution. No significant autocorrelation was found for discharge of nutrients and freshwater values, according to analysis of the autocorrelation function (ACF) or partial autocorrelation function (PACF). An *α* value for Type I errors of 0.05 was applied. Tests were two-tailed in all cases, where appropriate. Exponential model coefficients (cf. Eqn (2) and degree of determination was estimated by the curve estimation regression module in SPSS (v. 18). Model II linear regression was performed with a reduced major axis loss function and bootstrap estimate of coefficient standard error. Pearson or Spearman correlations were used, depending on the distribution of data. Choice of linear or nonlinear models was based on maximizing *R*^2^ and lack of dependence of residuals on the predicted values.

A cubic curve fit was applied using the SPSS® Curve estimation module to verify that the interannual dynamics with time (*t*) were significant, according to the model


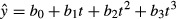
(1)

Data sets which lagged 1 and 2 years were analyzed. The residuals were tested for autocorrelation in the SPSS® Forecasting module and random distribution by graphic plots. Where significant autocorrelation was observed, data were also applied to an autoregressive integrated model (ARI) with appropriate lag and difference of two. Degree of explanation, (*R*^*2*^), root mean squared error (RMSE) and other model quality measures in the SPSS® Fit syntax were used to assess the best model. Cubic curve models showed the best fit to observed data, except for P_b_:P_p_ vs. time in the Bothnian Sea, where an ARI (4,2) model was used.

A combined standard uncertainty equation was applied to estimate the relative contribution of bacterial and phytoplankton community biomass production, respectively, to the change in P_b_:P_p_ ratio, as outlined in the published guide to the expression of uncertainty in measurement (GUM, b^2008^, Eq. 10).

### Data storage

All marine data are archived in the regional database dBotnia at Umeå Marine Sciences Centre (http://www.umf.umu.se) and the Swedish national marine database at the Swedish Meteorological and Hydrological Institute (SMHI).

## Results

**Fig. 2:**
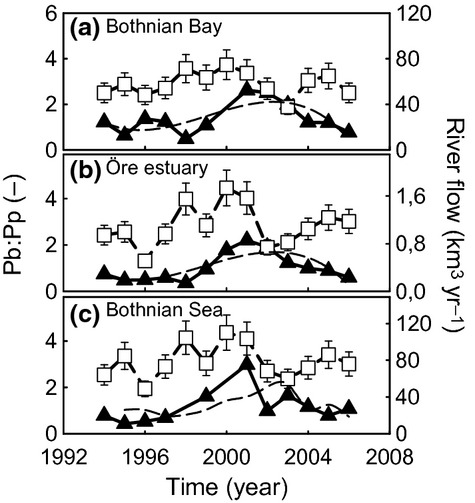
The ratio between bacterioplankton biomass production (P_b_) and phytoplankton biomass production (P_p_) vs. time (filled triangles). Non-linear model fits (dashed line) suggested that the trends were significant in all cases (*R*^*2*^ > 0.40, *P* < 0.037). An autoregressive, integrated model was used in the Bothnian Sea. River freshwater flow to the basin is also shown (open squares). Error bars show the summarized standard deviation of annual values based on 5% error of monthly means. The total number of samples per year was 72–96 yr^−1^.

The annual precipitation showed an elevated level between 1998 and 2001 over the drainage and sea area for both the Bothnian Bay and Bothnian Sea (Fig. S2). The value for year 2000 in the latter area (846.6 mm) was the highest precipitation recorded since 1930 (SMHI, b^2009^). A natural pulse of freshwater discharge consequently occurred during the years 1998–2001 in northern Scandinavia, when the discharge was higher than in adjacent years (Fig. 2; Table 2). The freshwater input was 1.3–1.8 times higher during this high-flow period than during 1994–1997 (ANOVA Bonferroni *post hoc* test, *P* < 0.033 in all cases, *n* = 4). For the Bothnian Sea in the northern Baltic, the flow rates for three out of these 4 years were close to the highest recorded in a 40 year time series, with a peak value 21% higher than the long-term average (91 km^3^ yr^−1^) (Bergstrom & Carlsson, b^1994^).

**Table 2:** Statistics of freshwater and nutrient discharge to the sea basins. Data is aggregated in symmetrical time periods to allow statistical tests. Molar ratios of the nutrients are shown. The probability of a Type I error for difference between periods (given by the letters) is shown (*p*). A univariate ANOVA test with Bonferroni's *post hoc* test for multiple comparisons for equal variances was used

	Period mean			*P*		
		
	1994–1997 (a)	1998–2001 (b)	2002–2006 (c)	a vs. b	b vs. c	a vs. c
Bothnian Bay						
River flow (km^3^ yr^−1^)	52	70	53	0.033	0.032	1
Total C (10^6^ kg yr^−1^)	258	440	283	0.019	0.033	1
Total N (10^6^ kg yr^−1^)	15	24	17	0.009	0.24	1
Total P (10^6^ kg yr^−1^)	1.0	1.1	0.74	1	0.156	0.334
C:P	678	1020	978	0.005	1	0.008
C:N	19	22	19	0.405	0.204	1
Öre estuary						
River flow (km^3^ yr^−1^)	0.85	1.5	0.98	0.011	0.03	1
Total C (10^6^ kg yr^−1^)	8.8	18	11	0.022	0.081	1.000
Total N (10^6^ kg yr^−1^)	0.35	0.61	0.44	0.018	0.114	0.688
Total P (10^6^ kg yr^−1^)	0.028	0.034	0.022	1	0.661	1
C:P	1072	1354	1349	0.783	1	0.738
C:N	29	33	29	0.263	0.189	1.000
Bothnian Sea						
River flow (km^3^ yr^−1^)	68	99	53	0.033	0.032	1.00
Total C (10^6^ kg yr^−1^)	375	663	424	0.008	0.017	1.00
Total N (10^6^ kg yr^−1^)	22	35	24	0.007	0.014	1.00
Total P (10^6^ kg yr^−1^)	0.92	1.3	0.70	0.097	0.006	0.45
C:P	1085	1338	1571	0.33	0.36	0.016
C:N	20	22	20	0.36	0.55	0.93

Total organic carbon (TOC_r_) and total nitrogen (TN_r_) load showed a strong correlation with flow rate, explaining the major part of their interannual variation (linear regression, *R*^2^ > 0.92, *P* < 0.001 for all basins, *n* = 13) (On-line data base 2009). The flow rate also explained a large but somewhat smaller part of the variation in total phosphorus (TP, *R*^2^ > 0.40, *P* < 0.021). The variation in flow rate in Fig. 2 therefore also indicates the major variation in load of TOC_r_, TN_r_, and to some extent TP_r_ to the marine environment.

The load of TN_r_ and TOC_r_ was significantly higher in all basins during 1998–2001 than in the preceding period (*P* > 0.018, ANOVA with Bonferroni's *post hoc* test, Table 2). For TP_r_, only the *P*-value for the Bothnian Sea (0.097) approached the applied significance level. Nonetheless, the mean value of *P* load during 1998–2001 was 7.7%, 21%, and 41% greater than the preceding period, in the Bothnian Bay, Öre estuary, and Bothnian Sea, respectively. The result showed that interannual variability in P load was too great to be able to detect statistically this magnitude of difference. No significant change in the C:N ratio between the periods could be shown. However, the C:P ratio of discharged material increased during the studied period by about 50% in the Bothnian Bay and Bothnian Sea (Bonferroni test, *P* = 0.005, *n* = 4), while the 26% increase in the Öre estuary could not be shown to be significant (*n* = 4).

We demonstrated a significant shift in the ratio between bacterioplankton biomass production and phytoplankton biomass production (P_b_:P_p_, i.e., ‘trophic balance’) associated with the freshwater pulse (Fig. 2; Table S1). A cubic curve fit verified a statistically significant peak after year 2000 in the Bothnian Bay (*R*^*2*^ = 0.60, *P* = 0.034) and the Öre estuary (*R*^*2*^ = 0.67, *P* = 0.014), while an autoregressive ARI (1, 1) model was significant in the Bothnian Sea (*R*^*2*^ = 0.40, *P* = 0.037). The first derivative at value zero suggested the maximum was at year 2002 at all sites (graphic determination for the ARI model). A lag of 2 years between river discharge and response in P_b_:P_p_ ratio was indicated in the time series (Fig. 2). Comparing 1994–1997 to 1998–2001, the relative importance of microbial heterotrophic productivity increased in all studied sites by a factor of 1.3–2.6. Five years after the end of the high flow period (2004–2006), the P_b_:P_p_ ratio was still skewed towards heterotrophy in the Bothnian Sea (Table S1, *t*-test, *t* = −2.82, df = 4, *P* = 0.048). In the Bothnian Bay and Öre estuary basins, the P_b_:P_p_ ratio was not significantly different during 2004–2006 compared to the level before the flow peak.

Maximum and minimum values of P_b_:P_p_ had a ratio of between five and seven depending on the site, showing that variation in trophic balance in the coastal ecosystem may be substantial (Table S1). However, the interannual variability in the P_b_:P_p_ ratio appeared steadier before the peak flow, independent of site (±CV = 25%, 1994–1997). Consequently, even a relatively modest increase in river flow, amounting to 32–76% of the 1994–1997 rate (albeit +21% of the long term average), had a major effect on the coastal P_b_:P_p_ ratio in our case study.

The importance of microbial heterotrophic processes (P_b_:P_p_) increased exponentially with increased discharge of riverine TOC to the coastal zone (TOC_r_), when lagged +2 years according to the model


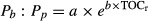
(2)

This was valid for all sites, based on statistically significant coefficients from an exponential regression (Fig. S3, Table 3). TOC_r_ explained a significant part of the variation (i.e., *R*^2^) in P_b_:P_p_ at all sites, with the highest value at the coastal site. The y-intercepts were significant in the Bothnian Bay and Öre estuary. Only the *b* coefficient in the Öre estuary was different from the off-shore basins, based on 2 × SE, a consequence of the higher TOC_r_ to this coastal site. A model II linear regression also showed significantly positive slope coefficients (data not shown).

**Table 3:** Statistics of exponential curve fit regression for the relationship between P_b_:P_p_ and specific TOCr discharge (lag = year + 2). The Y-intercept (a), exponential coefficient (b) and their standard error (SE), probability of type I error (*P*), and number of annual values analyzed (*n*) are shown. One missing P_b_:P_p_ value in each off-shore basin was interpolated

	(−)			([g C]^−1^ m^2^ yr)			
			
Basin	a	SE (a)	*P* (a)	b	SE (b)	*P* (b)	*n*
Bothnian Bay	0.56	0.23	0.035	0.093	0.041	0.049	11
Öre estuary	0.36	0.12	0.015	0.0050	0.0016	0.013	11
Bothnian Sea	0.26	0.17	0.15	0.18	0.081	0.053	11

Most of the change in P_b_:P_p_ was due to a decrease in phytoplankton biomass production, rather than an increase in bacterioplankton biomass production (Fig. S1). A variance component analysis (standard uncertainty calculation) suggested that, on average, 71% of the variation in the P_b_:P_p_ ratio was due to the decrease in phytoplankton biomass production and 29% was due to increase in bacterioplankton biomass production (Table S2). The difference between minimum and maximum phytoplankton annual production was also unexpectedly large, threefold to sixfold depending on sea basin (Fig. S1). However, where more freshwater discharge promoted increased input of TN_r_ and TP_r_, and thus potentially increased phytoplankton production, our data showed that it instead decreased (Fig. S1; Table 2).

**Fig. 3:**
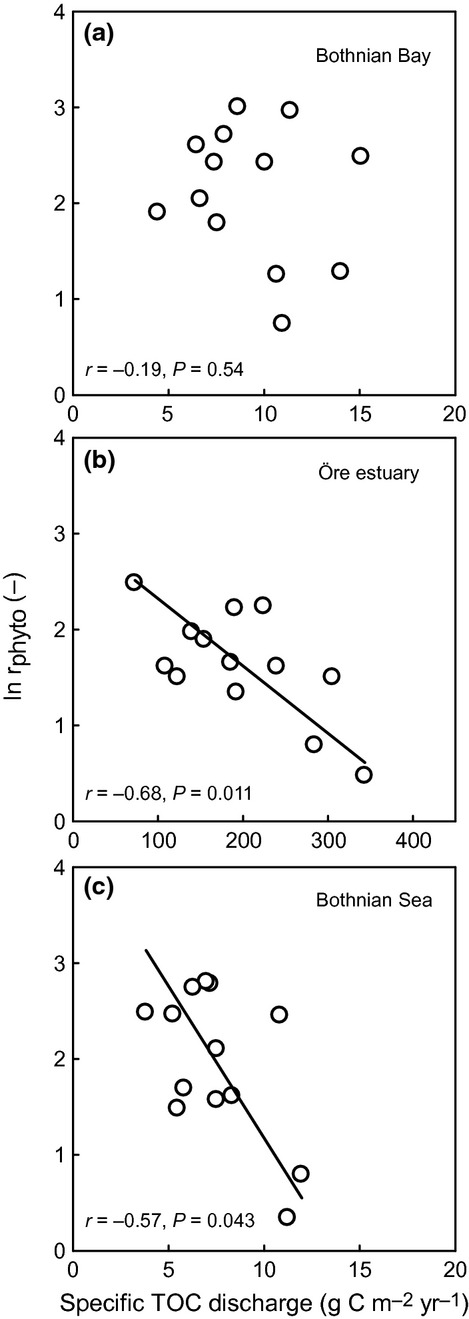
Phytoplankton specific growth rate (r_phyto_) is shown as a function of the specific riverine TOC discharge. The line of natural logarithm transformed values was derived using a Model II linear regression with a major axis loss function. The slope coefficient for the Bothnian Bay had a *P* value > 0.05.

To test for a potential effect on phytoplankton due the change in growth conditions, *r*_phyto_ was analyzed with specific TOCr discharge by a model II regression and Pearson correlation (Fig. 3). This showed a significant negative relationship for both the Öre estuary (*P* < 0.05, *R*^2^ = 0.46) and the Bothnian Sea (*P* < 0.05, *R*^2^ = 0.32). The Bothnian Bay did, however, not show a significant correlation, while a negative slope coefficient was demonstrated (*P* < 0.05).

## Discussion

**Fig. 4:**
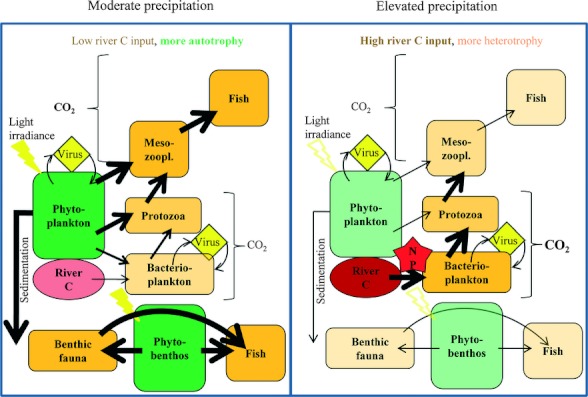
Proposed explanatory model for the observed carbon flows in the coastal food web at moderate and elevated precipitation scenario, respectively. Dense filling shows high light irradiance to phototrophic organisms and abundance of trophic groups, respectively. Thick arrows or text denote higher carbon flow rate and thin lower between trophic compartments. Star with ‘N P’ illustrates intensified competition between phyto- and bacterioplankton for inorganic nutrients. Higher respiration of carbon dioxide is indicated by bold face CO_2_. See discussion for further explanation.

Increased precipitation around the millennium shift caused a marked increase in freshwater discharge during a few years, and also direct input of freshwater and nutrients to the sea surface by rain (Figs 3 and 4). By using this extreme peak flow of river water in a 40 year time series, in conjunction with long-term ecological marine time series data, we demonstrated a marked shift from autotrophy (P_b_:P_p_ < 1) to heterotrophy (P_b_:P_p_ > 1) in three coastal sites (Fig. 2; Table S1). At all sites, the P_b_:P_p_ ratio was positively correlated with the load of TOC_r_ (Fig. S3; Table 3). This result showed that the net effect of increased freshwater flow to the marine environment hampered phytoplankton biomass production, while bacterial biomass production was maintained. This observation was unexpected considering that increased river flow was accompanied by an increase in TN_r_ and TP_r_, implying increased phytoplankton biomass production on mineralized nutrients. However, the observed effect may be explained by including influence of freshwater, TOC_r_, and bacterioplankton growth in production models for the coastal ecosystem, as outlined below.

The discharge of riverine TOC was a major driver of the P_b_:P_p_ ratio at all sites, assuming some contribution to the remaining variation from random measurement variance (*R*^2^-values, Fig. S3). The slope coefficients were not significantly different between the off-shore basins, suggesting a similar response on trophic balance from increased TOCr discharge. The markedly lower slope coefficient for the coastal Öre estuary reflects the markedly higher specific load of riverine TOC at this coastal site. However, due to the correlation between TOC_r_ and freshwater discharge (*R*^2^ > 0.92), either factor may still contribute to explain the observed change in trophic balance. The degree of explanation was slightly lower in the off-shore basins when using freshwater discharge as the independent variable, and the slope coefficient for the Bothnian Sea was not significantly different from zero. This indicates a specific importance of discharged TOCr for the observed effects. Other variables investigated to explain the variability in the P_b_:P_p_ ratio, such as the C:P ratio in river discharge, marine nutrient concentrations or global photosynthetically active irradiation, did not show significant correlation in any basin (data not shown). The only exception was marine inorganic nitrogen, which showed a positive relationship with P_b_:P_p_ in the Bothnian Sea (*r* = 0.80, *P* = 0.02).

Our results suggest that the suppression of phytoplankton biomass production explained a greater proportion (71%) of the change in P_b_:P_p_ ratio than an increase in bacterial biomass production (Table S2). Therefore, an explanation of the change in P_b_:P_p_ ratio should be primarily sought in the control of phytoplankton biomass production. One contributing reason for the lack of a positive response in phytoplankton biomass production was that increased freshwater flow mainly increased the TOC_r_ and TN_r_ load, while an increase in TP_r_ was moderate and not statistically significant (Fig. S1; Table 2). Thus, the increase in the major limiting nutrient, phosphorus, was lower relative to other nutrients, at elevated flows of freshwater. Phosphorus is considered the limiting nutrient in most of this study area, while a shift to nitrogen limitation occurs in the southern Bothnian Sea (Zweifel *et al*., b^1993^; Andersson *et al*., b^1996^). Total nutrient and carbon pools were used, as these also encompass organic and particulate fractions, assumed to be largely available also to osmotrophs by phagotrophy, mineralization, and photolysis in the food web during the long time scales investigated (Kieber *et al*., b^1990^; Sandberg *et al*., b^2004^).

The lag applied in Table 3 means that the TOCr discharge at a given year resulted in a full effect showing in the trophic balance 2 years later (Fig. 2). Based on the primary data, this is caused by the reduced bacterial biomass production in the first year of high freshwater discharge (year 1998, Fig. S1). The P_b_ then successively increased to preflow level or above, also elevating the P_b_:P_p_ ratio. A hypothesis to explain the observed lag period may be that initially recalcitrant carbon and nutrient substrates (organic and particle bound), are gradually made available by exudates released during phagotrophy of particles, mineralization, and photolytic cleavage of dissolved organically bound nitrogen and phosphorus, when exposed to the marine environment (Pengerud *et al*., b^1987^; Kieber *et al*., b^1990^; Thingstad *et al*., b^1993^). In addition a change in bacterioplankton diversity, as an adaptation to the newly available substrate resources, may gradually promote a more efficient metabolism of the terrigenous substrates, based on observations in culture studies (Kisand *et al*., b^2002^). The response of the phytoplankton specific growth rate (i.e., *r*_phyto_), in contrast, was immediate, as shown by a significant relationship with TOCr discharge, without lag, discussed below (Fig. 3).

Field data supported the hypothesis that growth limitation of phytoplankton was present because of a significant negative relationship between *r*_phyto_ and specific TOCr discharge (Fig. 3). The realized *r*_phyto_ is expected to decrease with enhanced competition, and thus, in this case, with increased P_b_:P_p_. The strongest influence of TOC_r_ (i.e., highest slope coefficient) occurred in the Bothnian Sea, suggesting this basin to be most sensitive to elevated freshwater discharge. The negative effect on *r*_phyto_ could, however, also result from other factors, e.g., reduced effective light climate as a consequence of discharged coloured and suspended matter, or changed stratification (Howarth *et al*., b^2000^; Hessen *et al*., b^2010^). This was based on that the results were almost identical when using freshwater discharge as the independent variable (data not shown). We can only speculate that the lack of a significant correlation in the Bothnian Bay was due to the smaller contribution of phytoplankton community growth to the observed change in trophic balance in this basin (Table S2).

The effect of freshwater discharge on phytoplankton growth without lag suggested an influence of factors in the discharge which exerted an immediate action (Figs 3 and S1). Discharge of coloured humic substance, suspended material and a deeper mixed layer, reducing the effective light climate for phytoplankton, are all in synergy and expected to act without a time lag on the annual scale (Cole *et al*., b^1992^; Howarth *et al*., b^2000^; Hessen *et al*., b^2010^). This explanation is also in accordance with model analyses, suggesting light limitation to override nutrient limitation in deeper and better mixed water columns (Jager *et al*., b^2010^). The simultaneously increased load of carbon to the coastal zone provided another, synergistic, explanation for the shift in carbon balance, and reduction in phytoplankton biomass production. Riverine dissolved organic carbon is an alternate carbon source of phytoplankton released carbon for the heterotrophic bacteria (Zweifel *et al*., b^1993^; Wikner *et al*., b^1999^). This may have promoted a competition between bacterio- and phytoplankton for inorganic nutrients, where the former has superior affinity for these substrates (Pengerud *et al*., b^1987^; Mindl *et al*., b^2005^). As bacterioplankton community growth showed a lag of 2 years, competition for limiting nutrients is, however, not likely to explain the initial reduction in phytoplankton growth (Fig. S1). When the bacterioplankton community with time became better able to exploit the more riverine-influenced carbon source the competition scenario may have contributed by adding a synergistic hampering effect on phytoplankton production. Simultaneous discharge of elements such as Fe associated with TOCr discharge may in addition, by chemical binding, have reduced the available pool of PO_4_^3−^ for phytoplankton (Blomqvist *et al*., b^2004^). Consequently, reduced access to mineral nutrients may also explain the hampered phytoplankton biomass production. The relative importance of changed light conditions for phytoplankton and competition of limiting nutrient was however not investigated in this study.

Similar studies of the composite effects of discharged carbon, nitrogen, and phosphorus on trophic balance in the coastal zone are scarce in the literature, especially given the datasets cover large temporal and spatial scales. In a long-term study of the Hudson estuary, Howarth *et al*. (b^2000^) stressed the importance of climate drivers, such as freshwater discharge, on primary production. Similar to our findings, primary production was suppressed at higher levels of freshwater discharge in their study, primarily explained by weaker stratification and light penetration. Caraco & Cole (b^2003^) also reported a tenfold lower ratio of de novo gross primary production to allochthonous organic load during a wet year than a dry one in the Hudson River, in agreement with our conclusions. In an estuary-lagoon system in eastern Brazil, increased freshwater flow was found to stimulate bacterial production while hampering primary production, with a resulting P_b_:P_p_ ratio of up to 20 during the rainy season (Barrera-Alba *et al*., b^2009^). Our findings were also in line with the ‘Thingstad-paradox’ observed in an Arctic mesocosm experiment, in which addition of carbon substrate led to a lower accumulation of carbon in the system, due to hampered phytoplankton biomass production (Thingstad *et al*., b^2008^). Similar results are reported from the Hunter Estuary, Australia, where addition of glucose reduced chlorophyll-*a* concentration, while increasing bacterial abundance and respiration (Hitchcock *et al*., b^2010^). In shallow unproductive lakes increased load of terrigenous dissolved organic carbon (DOC) is also responsible for hampering total lake productivity (Karlsson *et al*., b^2009^). Thus, our results add support to an emerging picture of TOC availability as an important, but counterintuitive, controller of aquatic productivity. This study contributes by showing the importance of riverine TOC for the coastal zone at large temporal and spatial scales. It also provides evidence for a general relationship between the riverine TOC supply and P_b_:P_p_ ratio of the coastal sea.

The explanatory model outlined above seems mainly associated with estuaries at temporal scales from seasonal to annual. In short-term studies of freshwater plumes entering directly into oceanic waters (i.e., Strait of Georgia, Bay of Bengal), a stimulating effect on primary production has been reported (Harrison *et al*., b^1991^; Gomes *et al*., b^2000^). Effects were, however, reported to alternate between negative and positive, depending on the temporal scale in one particular environment. Thus, in oceanic environments positive effects of freshwater plumes on plankton productivity are possible, suggesting a lower negative influence of the freshwater and TOC supply on phytoplankton growth in these environments.

We consider the bacterial and phytoplankton biomass production ratio to have sufficient accuracy when estimating the distribution of major pelagic carbon flows (i.e., microbial hetero- vs. autotrophic processes). Phyto- and bacterioplankton biomass production clearly dominates the marine food web carbon flow, and are therefore pivotal factors in coastal ecosystem function (Kemp *et al*., b^1997^; Sandberg *et al*., b^2004^). P_b_:P_p_ values have further been shown to give similar results to alternative methods using mass balances or oxygen models in estimating net ecosystem metabolism, net CO_2_ emission and net heterotrophy (Stigebrandt, b^1991^; Kemp *et al*., b^1997^; Sandberg *et al*., b^2004^). It is also well documented that increase in bacterial biomass production will promote carbon flow through the multi-trophic level protozoan dominated food chain, with associated respiration losses (Pengerud *et al*., b^1987^; Wikner & Hagström, b^1988^; Sandberg *et al*., b^2004^; Berglund *et al*., b^2007^). Lower fish production and sedimentation is thereby expected, compared to the shorter food chain associated with the flow of phytoplankton biomass directly through the mesozooplankton community. Bacterio- and phytoplankton biomass production are therefore key processes for the trophic balance and food web efficiency of the coastal sea.

The found relationship between riverine discharge of TOC and coastal trophic balance suggests that increased precipitation, and the resulting higher load of freshwater and terrigenous TOC, will lead to a food web dominated by heterotrophic microorganisms (Sandberg *et al*., b^2004^; Berglund *et al*., b^2007^; Fig. 4). As production of organic matter by phytoplankton is simultaneously suppressed, a lowered supply of food to fish and benthic organisms is expected (Nixon, b^1988^; Berglund *et al*., b^2007^; Finkel *et al*., b^2010^). Our view of the effect of precipitation on coastal productivity is thus opposite to recent assessments of climate-change effects for the Baltic Sea (Neumann, b^2010^). Neumanns's model study suggested an increase in phytoplankton biomass of 5% and extended growth season at elevated precipitation based on the classical view of phytoplankton growth limited only by phosphorus or nitrogen. Influence of freshwater discharge on pelagic stratification, light climate, and concentration of riverine TOC should therefore be included in current models for coastal management and resource utilization (e.g., mitigation of eutrophication and allowable fish catch), as well as in forecasting the consequences of climate change (Fig. 4). Supply of nitrogen and phosphorus alone are insufficient to accurately predict coastal productivity.

## Supporting Information

**Figure S1.** Annual phytoplankton- and bacterioplankton biomass production rate during the investigation period.

**Figure S2.** Annual precipitation at representative stations in the Bothnian Bay and Bothnian Sea drainage areas, respectively.

**Figure S3.** Trophic balance (P_b_:P_p_-ratio) as a function of specific TOC discharge with a 2 year lag.

**Table S1**. Statistics and comparison of the P_b_:P_p_ ratio between time periods (a–c).

**Table S2.** Variance contribution from phytoplankton (Vp) and bacterioplankton biomass production (1-Vp), respectively, to the variation in the Pb:Pp ratio (VB:P).
