# Supplementary material for: Increased freshwater discharge shifts the trophic balance in the coastal zone of the northern Baltic Sea
Source: Glob Chang Biol. 2012 May 17;18(8):2509–19. doi: 10.1111/j.1365-2486.2012.02718.x (PMC3597252; doi:10.1111/j.1365-2486.2012.02718.x)
Supplement: Supplementary file 1 [file gcb0018-2509-SD1.docx]

Supporting information
“Freshwater causes marine trophic shift”

Supporting Figures

Figure S1

 Fig. S1. Phytoplankton- (filled circles) and bacterioplankton biomass production rate (open triangles). Values were integrated over the average depth of the basin for bacterioplankton biomass production rate and over 20 m depth for phytoplankton biomass production rate. A cubic curve model (dashed line) suggested the overall trend in phytoplankton biomass production to be significant in all sites (*R^2^*>0.65*, p*<0.020 in all cases). None of the sites showed significant cubic trend for bacterioplankton biomass production (*p*>0.42). The total number of samples per year was 72–96 yr^-1^.

Figure S2

Fig. S2. Annual precipitation at representative stations for the Bothnian Bay and Bothnian Sea areas, respectively.

Figure S3

Fig. S3. Trophic balance (P_b_:P_p_) is shown as a function of the specific riverine TOC discharge with +2 years lag. The solid line show predicted values according to an exponential model (Eq. 2).

Supporting Tables

Table S1. Statistics and comparison of the P_b_:P_p_ ratio between time periods (a-c). Mean values prior to, during and after the high flow period are shown. Mean, maximum (max.) and minimum (min.) values for the whole period are also presented. *φ*TOCr shows the fraction of riverine TOC in the major coastal carbon supply (mean whole period, ±*SD*).

|  | Mean a | Mean b | Mean c | Min. | Mean | Max. | Max.:Min. | Mean b: Mean a |  |
| --- | --- | --- | --- | --- | --- | --- | --- | --- | --- |
| Location | 1994-1997 | 1998-2001 | 2002-2006 | whole period | | |  |  | *φ*TOC_r_ (%) |
| Bothnian Bay | 1.1 | 1.4 | 1.4 | 0.49 | 1.36 | 2.62 | 5 | 1.3 | 43 ±14 |
| Öre Estuary | 0.58 | 1.3 | 1.1 | 0.35 | 1.02 | 2.20 | 6 | 2.2 | 24 ±13 |
| Bothnian Sea | 0.61 | 1.6 | 1.1 | 0.43 | 1.07 | 2.98 | 7 | 2.6 | 22 ±15 |

Table S2. Variance contribution from phytoplankton (Vp) and bacterioplankton biomass production (1-Vp), respectively, to the variation in the Pb:Pp ratio (VB:P).

| Basin | V_B:P_ | V_P_ |
| --- | --- | --- |
|  | (-) | (%) |
| Bothnian Bay | 0.38 | 62 |
| Öre estuary | 0.14 | 75 |
| Bothnian Sea | 0.21 | 74 |
| All areas | 0.48 | 71 |
